# Supplementary material for: Chromothripsis during telomere crisis is independent of NHEJ, and consistent with a replicative origin
Source: Genome Res. 2019 May;29(5):737–49. doi: 10.1101/gr.240705.118 (PMC6499312; doi:10.1101/gr.240705.118)
Supplement: Supplemental Material [file supp_gr.240705.118_Supplemental_file_1.zip › contigs/annotated_contigs/DB55/contig.2.DB55_length_686_mean_cov_3.71720116618.docx]

**DB55_length_686_mean_cov_3.71720116618**

```
GTCAGGAAAGCTGGGTACAGATGTTGGTGGCGCACTTTCTTTGTGATCTATAGGAAGTACTCACCTGTACAGTCTGTTTCCTCACCAGC
 >chr7:150855615-150856099 - E=1e-279 p=3e-02
AAAATAAGGACAGTAATATTTACCTCACAGGGTTAAGGATTAAATGCACAGATATTTGTGCAGCATCCTGAAAGTAATAGGTGCTCAAG

AATTTATCCTTATTGTCCTTTTCTTGGAAATATGTGAGGCGCTTTCCTGGGAATTAAGTTGGGAAAGATCTTGTTACTAGGAATGGTGT

AGGATAGGTGACACTCTATGTATTGAAAAGGACAAATATTTCTGGGAGTGATGATAGAGTAGTTCTGTGTTCTGAGAACTGATTATTCG

GGGACTGCCAGGAAAGAACATTGTACTGAATCTAATTAGGGGTAGATTTCACATGTGTCTTTGGCTCTTTCTCCCTCTTTTCGGTTTCC

CTGTCTCACATTTTGGTGTCCTCTGTAAATCCCAAG|CCT|TTCCTTCTCTTCATTTGTCCCTGTTGCACTTTTCCCTCTTCCTCCTCC
                                     >chr7:150846974-150847179 - E=1e-111
TCCTCCCATATTCCTTCCAGTCCTCCCAGCTGACATCAAGCTTAGGGGCCTCAGGATTCTGAACTTGAGCAGAAAACAGCTGTTTGTGC

TGCTCAGGCATCCTTGGCTGGGGAAAGGGGAAGGTGGGAAAGAGATGGTTTCATTTTAAGGTCAA
```
